# Metabolomic differences between invasive alien plants from native and invaded habitats

**DOI:** 10.1038/s41598-020-66477-w

**Published:** 2020-06-16

**Authors:** Sarah A. Skubel, Xiaoyang Su, Alexander Poulev, Llewellyn C. Foxcroft, Vyacheslav Dushenkov, Ilya Raskin

**Affiliations:** 1 Department of Plant Biology, Rutgers, The State University of New Jersey, New Brunswick, New Jersey United States of America; 2 Department of Medicine, Robert Wood Johnson Medical School, Rutgers, The State University of New Jersey, New Brunswick, New Jersey United States of America; 3 Conservation Services, South African National Parks, Skukuza, South Africa; 4 Centre for Invasion Biology, Department of Botany and Zoology, Stellenbosch University, Stellenbosch, South Africa; 5 Hostos Community College, City University of New York, Bronx, New York United States of America

**Keywords:** Metabolomics, Environmental sciences, Climate-change ecology, Ecosystem ecology, Evolutionary ecology, Invasive species, Speciation, Climate change

## Abstract

Globalization facilitated the spread of invasive alien species (IAS), undermining the stability of the world’s ecosystems. We investigated the metabolomic profiles of three IAS species: *Chromolaena odorata* (Asteraceae) *Datura stramonium* (Solanaceae), and *Xanthium strumarium* (Asteraceae), comparing metabolites of individual plants in their native habitats (USA), to their invasive counterparts growing in and around Kruger National Park (South Africa, ZA). Metabolomic samples were collected using RApid Metabolome Extraction and Storage (RAMES) technology, which immobilizes phytochemicals on glass fiber disks, reducing compound degradation, allowing long-term, storage and simplifying biochemical analysis. Metabolomic differences were analyzed using ultra-performance liquid chromatography-mass spectrometry (UPLC-MS) of samples eluted from RAMES disks. Partial Least Squares-Discriminant Analysis (PLS-DA) of metabolomes of individual plants allowed statistical separation of species, native and invasive populations of each species, and some populations on the same continent. Invasive populations of all species were more phytochemically diverse than their native counterparts, and their metabolomic profiles were statistically distinguishable from their native relatives. These data may elucidate the mechanisms of successful invasion and rapid adaptive evolution of IAS. Moreover, RAMES technology combined with PLS-DA statistical analysis may allow taxonomic identification of species and, possibly, populations within each species.

## Introduction

Invasive alien species (IAS) are a threat to global biodiversity and stable ecosystem function^1^. IAS colonize regions they are not native to, with the potential to disrupt the natural state of ecosystems^1^. An increase in IAS is observed in areas with high Human Development Index (HDI)^2^, correlating their spread to humans activities and urbanization^3^. The advancement of globalization has significantly contributed to IAS spread^3,4^, costing the United States (US) on average $8 billion in damages and environmental loss^5^. Similarly, in South Africa (SA), about $69 million per year is being spent on the management of invasive alien plants^6,7^. The successful invasion of alien plants is, in part, attributed to favorable resource conditions, weak or low competition and biological flexibility^8^. Native species co-evolved with natural pathogens and herbivores that control their density and distribution. Often the explosive spread of IAS suggests the existence of adaptive mechanisms that provide an advantage over native species and greater resistance to disease and herbivory. It is tempting to speculate that some of these mechanisms may involve biochemical flexibility that allows better adaptation to different habitats.

Earlier reports showed that plants with invasive tendencies have greater diversity of phytochemicals over non-native species^9^, suggesting that IAS diminish ecological diversity because of their superior biochemical flexibility^4,10^. However, we are not aware of any studies that directly compare metabolomic profiles of native and invasive population of the same species. Comparative analysis of metabolomic profiles of different IAS populations using modern analytical and computational techniques, may provide insight on how IAS adapt to foreign environments, and help to understand the evolutionary mechanism responsible for invasive behavior.

Biochemical adaptations affect competitive plant survival. Plants, as sessile organisms, respond to their environment by making a great diversity of secondary metabolites (plant natural products), protecting them from biotic and abiotic stresses, facilitating nutrient acquisition and attracting pollinators^11–13^. In contrast, mobile animals, inferior in their biosynthetic machinery, respond to stresses with a “fight or flight response”. Phytochemical diversity of plant metabolites represent years of evolutionary adaptation to environment, resulting in the production of specific metabolites as a response to particular stimulus and/or stress^11,14^.

We selected three plant species native to the US that are labeled invasive and pose severe ecological threats in South Africa’s largest protected area (~2 million ha), Kruger National Park (KNP). These plants are *Chromolaena odorata* L. King & H. Rob., (blue mistflower, Asteraceae family), *Datura stramonium* L. (jimsonweed, Solanaceae family), and *Xanthium strumarium* L. (common cocklebur, Asteraceae family)*.* We used RAMES technology as a field-deployable, rapid metabolome collecting method^15^. RAMES uses a fast and compact sampling, grinding and extraction process thereby extracts are sorbed onto 10 mm-wide glass fiber discs and air dried within minutes. Only 2 grams of tissues is harvested and extracted in ethanol resulting in 20–30 RAMES discs replicates that can be stored in a −20 °C freezer and subsequently eluted for UPLC-MS analysis. This method minimizes compound degradation and solves the issue of standardization across different collections.

Through collecting metabolomic samples from plants in their native (US) and invasive (SA) ranges, we aimed to evaluate whether invasive plants adapt their secondary metabolism to provide better defense against unique biotic and abiotic stresses encountered in the invaded habitats. We hypothesized that plants in the invaded habitats show metabolomic differentiation from their native habitats, and that analytical and statistical methods we developed are sensitive enough to differentiate species, and, possibly, geographically separated populations within each species. The goal of this research was to understand how plant metabolomic profiles relate to invasive behavior and adaptive evolution, and whether they could provide a new approach to taxonomic identification.

## Materials and Methods

### Species background and habitats

*C. odorata* is a bush-like, climbing perennial^10,16,17^, dispersing seed by wind and is a fast growing plant even during the dry season^16^. It is often found in disturbed areas, especially by riverbanks and agricultural areas^18^. Five continents have been invaded by this plant, with some speculation that *C. odorata* evolved different climatic requirements on some continents^19^. *D. stramonium*, is found in disturbed environments, and is a major agricultural weed^14^. It contains tropane alkaloids present in other members of the Solanaceae family^14,20–22^. Major tropane alkaloids in *D. stramonium* leaves are scopolamine and hyoscyamine^14,21^, with younger leaves having the highest concentrations of both compounds^21^. However, alkaloid content varies in different environments, possibly as an adaptive defense mechanism^23^. *X. strumarium*, an aggressive IAS and an agricultural weed, is an annual^24^ containing caffeoylquinic acid, carboxyatractyloside, and sesquiterpene lactones called xanthanolides, related to the antimalarial compound artemisinin^24^. It also contains carboxyatractyloside, a toxic diterpene, present in high concentrations in young plants^25^. *C. odorata* plants were first recorded in KNP in 1997^7^, *Xanthium* spp. were first recorded in 1953, and *Datura* spp. were first recorded in 1953^7^. The times at which these species invaded SA are not properly recorded, and likely exceed 150 years.

### Sampling locations and tissue collection

United States samples were collected under a permit granted by the New York Parks & Recreation Natural Resources Group, 2018, and with permission from Bok Tower Gardens, Florida. South African species were collected under permit number RASI1343 granted by KNP. *C. odorata* was sampled at four locations in each country, collecting three individuals per location. *D. stramonium* was sampled at three locations in US and SA, with three plants collected at each location. *X. strumarium* was sampled at three locations per country, with four individuals per location. At each collection site GPS coordinates, time of collection, weather, topography and descriptive characteristics of physiology on each sample plant was recorded in addition to comprehensive photo documentation (see Supplementary Table S1). Collections were performed under supervision of local botanists. In the US, *C. odorata* was identified by Dr. Greg Kramer, *D. stramonium* and *X. strumarium* were identified in field by Ms. Jessica Hoch. KNP species were identified by the author (LCF). We collected the youngest, fully developed leaves from each plant, presumed to have the highest concentrations of secondary metabolites.

### RAMES methodology

RAMES technology was used for metabolite extraction and storage as described in^15^. Two grams of leaf tissue, weighed on a portable electric balance (CS Series, Ohaus, Parsippany, NJ), was ground in 5 ml of 95% ethanol using a Dremel tool (Model 8220, division of Robert Bosch GmbH Co., Racine, WI), equipped with a specially designed extraction bit. Extracts were filtered and loaded onto 10 mm-wide Whatman glass microfiber filters, Grade GF/D (Whatman # 1823-010, purchased from Millipore Sigma), dried using a cordless fan (Efluky Mini USB 3 Speeds Rechargeable Portable Table Fan, 4.5-Inch) and stored in 50 mm × 50 mm zip-lock plastic bags at −20 °C within an hour of grinding and within 4 hours of initial tissue collection.

### Ultra-performance liquid chromatography-mass spectrometry analysis

For metabolomic analysis, RAMES discs were eluted with 95% ethanol in 20 ml scintillation vials and left on a shaker at 120 rpm overnight. All samples were standardized for a final concentration of 5 mg dry extract/ml and 1 μl from each sample was injected for analysis. Each sample was injected twice, one run in negative ionization mode of the MS, and another with positive ionization. There were 3 individual samples from each location both in the US and in South Africa. Two control samples were included with each sample set; one of the solvent, and another one with a blank disk eluate. UPLC/MS analysis was performed as described^15^ using the Dionex UltiMate 3000 RSLC ultra-high pressure liquid chromatograph, workstation equipped with the ThermoFisher Scientific’s Xcalibur v. 4.0 software package combined with Dionex’s SII LC control software, solvent rack/degasser SRD-3400, pulseless chromatography pump HPG-3400RS, autosampler WPS-3000RS, column compartment TCC-3000RS, and photodiode array detector DAD-3000RS. After the photodiode array detector the eluent flow was guided to a Q Exactive Plus Orbitrap high-resolution high-mass-accuracy mass spectrometer (MS) (Thermo Scientific, Waltham, MA). Mass detection was a full MS scan from 100 to 1000 *m/z* in either positive, or negative ionization mode with electrospray (ESI) interface. Sheath gas flow rate was 30 arbitrary units, auxiliary gas flow rate was 7, and sweep gas flow rate was 1. The spray voltage was 3500 volts (−3500 for negative ESI) with a capillary temperature of 275 °C. The mass resolution was 140,000 m/∆m FWHM. Compounds were separated on a Phenomenex Kinetex C8 reverse phase column, size 100 × 2.1 mm, particle size 2.6 µm, pore size 100 Å. The mobile phase consisted of 2 components: Solvent A (0.5% ACS grade acetic acid in LCMS grade water, pH 3–3.5), and Solvent B (100% Acetonitrile, LCMS grade). The mobile phase flow was 0.20 ml/min, and a gradient mode was used for all analyses. The initial conditions of the gradient were 95% A and 5% B; for 30 min the proportion reaches 5% A and 95% B, which was kept for the next 8 minutes, and during the following 4 min the ratio was brought to initial conditions. An 8 min equilibration interval was included between subsequent injections. The average pump pressure using these parameters was typically around 3900 psi for the initial conditions^15^.

### Metabolomics data processing and statistical analysis

Metabolite features were chosen in each plant species for quantitation. Each of the chosen features are a combination of accurate mass-to-charge ratio (*m/z*) and retention time (RT) and represents a well-known metabolite, isomer of well-known metabolite, or an unknown but highly abundant metabolite in that species. Since the aim of this study was to identify qualitative and/or quantitative differences of metabolites present in the US and SA plants based mainly on known metabolites, or unknown metabolites with relatively high concentration, no MS/MS data was collected to fully confirm the identities of all metabolites. To assign putative metabolite annotation to the features, we first determined the putative molecular formulas by performing isotope abundance analysis on the high-resolution mass spectral data with Xcalibur v. 4.0 (Thermo Scientific, Waltham, MA) software and reporting the best fitting empirical formula. Database searches were performed using the Reaxys.com (RELX Intellectual Properties SA) and SciFinder (American Chemical Society). Databases were reviewed for compounds identified from the analyzed genera with molecular masses corresponding to the LC-FTMS data. Any matches were investigated by comparing the literature and the experimental data; putative compound assignments were made when matches were identified. Quantitation of the features was performed in MAVEN^26^ to get the peak intensity list. Peak intensity list was further processed with MetaboAnalystR^27^. Intensity data of each sample were normalized by the median, log transformed, and auto scaled. The normalized data were used to generate the PLS-DA plots and the heatmaps. The permutational multivariate analysis of variance (PERMANOVA) was performed in R using the vegan package and p-values were calculated with 10,000 permutations^26,27^.

## Results

**Figure 1:**
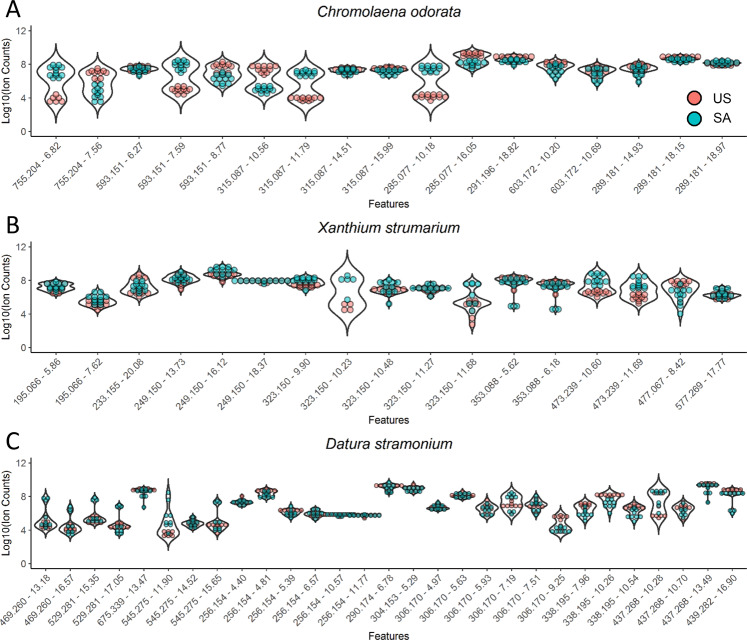
Distribution of the relative abundance of metabolites in *Chromolaena odorata* (**A**), *Xanthium strumarium* (**B**) and *Datura stramonium* (**C**). The intensity of a metabolite from each sample is represented as a colored dot in the plot. The violin plot of each metabolite shows the abundance distribution in the US (US) and South African (SA) samples.

A total of 17 secondary metabolites (including isomers) were putatively identified in *C. odorata* from the US and SA (Table 1). The relative abundance of secondary metabolites was compared in Fig. 1A using a violin plot that shows the relative abundance distribution for each metabolite. Some metabolites show more than three orders-of-magnitude abundance differences between US and SA samples. These include sakuranetin (*m/z* 285.077 [−ESI], RT 10.18 min) and dihydroxy-dimethoxy flavones (*m/z* 315.087 [−ESI], RT 11.79 min) suggesting large changes in the metabolomic profiles between continents. Many secondary metabolites were detected as multiple isomers that have the exact same *m/z* but were chromatographically resolved. Interestingly, some isomers showed opposite abundance distribution between US and SA samples. For example, the dihydroxy-dimethoxy flavones isomer with RT 10.56 min was more abundant in the US samples while the isomer with RT 11.79 min was more abundant in SA samples. Quercetin trisaccharide isomer with RT 6.82 min was more abundant in SA samples, while the isomer with RT 7.45 min was more abundant in US samples. These results suggest that, while the biosynthetic pathways of dihydroxy-dimethoxy flavones and quercetin trisaccharide are largely conserved from US to SA, the modification/substitution steps making different isomers vary significantly from one origin to another. Under ‘isomers’ different positional isomers are implied, e.g. hydroxyl and/or methyl, and/or methoxy group position in the ring structure of the compound. Ion source fragmentation ions indicated the most likely structure of the aglycone in the case of flavone glycosides. The exact same mass and fragmentation ion appearing at different RTs indicate different isomers of the same main structure. This is valid for all three species investigated in this study.

**Table 1:** Ion variants for *Chromolaena odorata* as determined by differing retention time calculations within mass to charge measurements.

	Name	*m/z* [M−H]^−^	Mol. formula	Retention time (RT, min)
1	Quercetin trisaccharides	755.204	C_33_ H_40_ O_20_	6.82
				7.45
2	Trihydroxyflavone (Kaempferol) disaccharides	593.151		6.27
			C_27_ H_30_ O_15_	7.59
				8.77
3	Dihydroxy-dimethoxy flavones	315.087	C_17_ H_16_ O_6_	10.56
				11.79
				14.51
				15.99
4	Isosakuranetin	285.077	C_16_ H_14_ O_5_	10.18
	Sakuranetin			16.05
5	Oxophytodienoic acid	291.196	C_18_ H_28_ O_3_	18.82
6	Flavone acetyl-disaccharides	603.172	C_29_ H_32_ O_14_	10.20
				10.69
7	Chromomoric acids	289.181	C_18_ H_26_ O_3_	14.93
				18.15
				18.97

All compound identification is putative and based on high mass accuracy mass spectrometry. For associated chromatograms see Supplementary Figs. S2 1,2.

**Figure 2:**
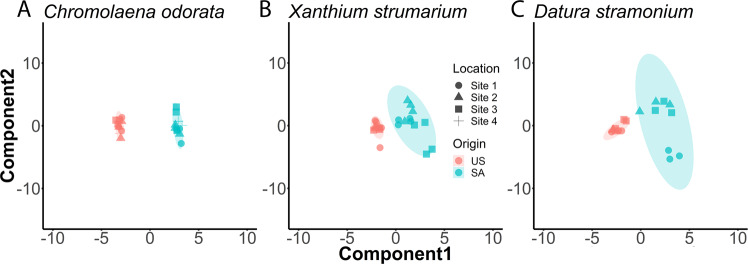
PLS-DA plots of metabolomic features in *Chromolaena odorata* (**A**)*, Xanthium strumarium* (**B**), and *Datura stramonium* (**C**). Each sample is represented as a symbol colored by origin. The shape of the symbol represents the sampling locations (Supplementary Table 1). The shaded area is the 95% confidence region of PLS component distribution.

**Figure 3:**
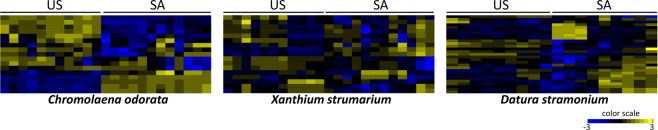
Heatmaps showing the comparison of the metabolomic profiles in *Chromolaena odorata* (left)*, Xanthium strumarium* (middle), and *Datura stramonium* (right). Each row representing a metabolomic feature is centered and unit variance scaled.

The metabolomes of *C. odorata* (11 from US and 12 from SA) were tested for similarity using permutational multivariate analysis of variance (PERMANOVA, Table 2). This analysis showed the US and SA plant metabolomes are statistically, significantly different (P = 0.0001). We further used PLS-DA score plot to visualize the different metabolomics profiles in US and SA samples (Fig. 2A). PLS-DA plots showed US and SA samples tightly clustered, suggesting samples from each continental origin share high similarity. US and SA samples, however, were well-separated on the PLS-DA score plot. A heatmap was also plotted to highlight the differences in the metabolomics profiles (Fig. 3). In the heatmap, metabolomic features having similar pattern are clustered together. The top features are more abundant in the US samples while the bottom features are more abundant in SA samples. There is a clear demarcation between the US and SA samples on the heatmap, suggesting a clear distinction in metabolomic profiles.

**Table 2:** PERMANOVA statistics of *Chromolaena odorata, Datura stramonium, and Xanthium strumarium*.

Species Name	Total df	F-stat	P-value
*Chromolaena odorata*	22	26.001	0.0001
*Datura stramonium*	17	4.1316	0.0018
*Xanthium strumarium*	23	5.2754	0.0001

A total of 17 different secondary metabolites (including isomers) were characterized in *X. strumarium* from the US and SA (Table 3), their relative abundance is compared in Fig. 1B. In general, the metabolite abundance in *X. strumarium* between the US and SA samples were more similar than in *C. odorata*. Nonetheless, metabolites such as akanthopyrone (*m/z* 473.239) showed higher abundance in SA samples than in US samples. The PERMANOVA test also revealed statistically significant differences in US and SA plant metabolomes (P = 0.0001, Table 2). We used PLS-DA score plots to visualize the different metabolomics profiles in US and SA samples (Fig. 2B). The US samples are closely clustered, much like *C. odorata*. One US sample is lying outside the 95% confidence region, suggesting it is an outlier. Indeed, this sample, US-26, is showing a different pattern than its neighbors on the heatmap (Fig. 3). The SA samples are all located in the 95% confidence region on the PLS-DA plot. However, the 95% confidence region of SA *X. strumarium* is much larger than 95% confidence region of SA *C. odorata*. This result demonstrates the greater metabolomic variability and diversity in the SA *X. strumarium*.

**Table 3:** Ion variants for *Xanthium strumarium* as determined by differing retention time calculations within mass to charge (*m/z*) measurements.

	Name	*m/z* [M−H]^−^	Mol. formula	Retention time (RT, min)
1	Dihydroferulic acid	195.066	C_10_ H_12_ O_4_	5.86
				7.62
2	Sesquiterpene lactones	233.155	C_15_ H_22_ O_2_	20.08
				20.82
3		249.150	C_15_ H_22_ O_3_	13.36
				13.73
				14.45
				14.74
				16.12
				18.36
4		323.150	C_17_ H_24_ O_6_	9.90
				10.48
				11.27
5	Chlorogenates	353.088	C_16_ H_18_ O_9_	5.62
				6.18
6	Akanthopyrones	473.239	C_23_ H_38_ O_10_	8.95–10.40 adducts/fragments
				10.60
				11.69
7	Quercetin glucuronide	477.067	C_21_ H_18_ O_13_	8.42
8	Sesquiterpenes; Sesquiterpene lactones	251.165	C_15_ H_24_ O_3_	15.33
				15.72

All compound identification is putative and based on high mass accuracy mass spectrometry. For associated chromatograms see Supplementary Figs. S2, 3,4.

A total of 29 different secondary metabolites (including isomers) were characterized in *D. stramonium* from the US and SA (Table 4). LC-MS analysis was performed under positive ionization mode to cover alkaloids and negative mode to cover other metabolites. Relative abundance of secondary metabolites were compared in Fig. 1C. In general, the metabolite abundance in *D. stramonium* between the US and SA samples were more similar than that in *C. odorata*. Nonetheless, metabolites such as ditigloyloxytropanol (*m/z* 338.194 [+ESI]) show higher abundance in US samples than in SA samples. Despite greater metabolomic variability, just as in other species, the PERMANOVA test shows statistically significant differences between US and SA populations (P = 0.0018, Table 2). PLS-DA score plot (Fig. 2C) showed US and SA samples separating without overlapping 95% confidence region. The confidence region of SA samples is larger than that of US samples, suggesting a high degree of variability in the metabolome of SA samples. PLS-DA score plots can also reveal within class variations. PLS-DA model is aware of the group labels but agnostic on the sample identity within a group. Therefore, the PLS-DA score plot is more reliable in demonstrating subgroup structures than assessing the separation of two groups. On the PLS-DA score plot of *D. stramonium*, three samples collected at SA site 1 (round symbols) are separated from other SA samples. In the heatmap (Fig. 3) of the *D. stramonium* metabolomics features, these three samples SA-55, SA-56 and SA-58 showed distinct patterns including elevated levels of withanolide derivatives (*m/z* 429.260 and 529.281 [−ESI]) and steroid lactone glycosides (*m/z* 545.275 [−ESI]). Our sampling records (Supplementary Table S1) show these three samples were from the collection site in the southern region of KNP, while the other samples were from collection sites in the northern region. These results suggest that micro-migration may also shift metabolomic profiles and that the methods of metabolomic collection and analysis used in this work are sensitive enough to separate different populations. The US samples of *D. stramonium* are generally clustered well on the PLS-DA plot. Two samples (square symbols) are more apart from other samples. These two samples were collected at a New Jersey site while other sample collection sites are in New York state. These results suggest that the micro-migration changes the metabolome more drastically in the IAS than the ones in their natural habitat.

**Table 4:** Ion variants for *Datura stramonium* as determined by differing retention time calculations within mass (*m/z*) to charge measurements.

	Name	*m/z* [M−H]^−^	Mol. formula	Retention time (RT, min)
1	Withanolide derivatives	469.260	C_28_ H_38_ O_6_	13.18
				16.57
2		529.281	C_30_ H_42_ O_8_	8.77
				15.35
				17.05
3	Cucurbitacin glycoside	675.339	C_36_ H_52_ O_12_	13.47
				11.90
4	Steroid lactone glycosides	545.275	C_30_ H_42_ O_9_	14.52
				15.65
	**Name**	***m/z*** **[M** + **H]**^**+**^		**Retention time (RT, min)**
5	Tigloyltropane derivatives	256.154	C_13_ H_21_ NO_4_	4.40
				4.81
				5.39
				6.57
				10.57
6	Atropine	290.174	C_17_ H_23_ NO_3_	6.78
7	Scopolamin	304.153	C_17_ H_21_ NO_4_	5.29
8	Hydroxy-hyoscyamine; Atropine N-oxide	306.170	C_17_ H_23_ NO_4_	4.97
				5.93
				7.19
				7.51
9	Tropane derivatives	320.149	C_17_ H_21_ NO_5_	4.96
				6.58
10	Ditigloyloxytropanols	338.195	C_18_ H_27_ NO_5_	7.96
				10.26
11	Daturilin/Withametelin derivatives	437.268	C_28_ H_36_ O_4_	9.48
				10.28
				13.49
12	Hydroxy-oxowithatrienolide	439.282	C_28_ H_38_ O_4_	16.90
13	Withadienolide deriv.	457.294	C_28_ H_40_ O_5_	9.25
				10.70
				11.77

All compound identification is putative and based on high mass accuracy mass spectrometry. For associated chromatograms see Supplementary Figs. S2 5–8.

## Discussion

Our results provide the first demonstration of the significant differences in the metabolomic profiles between IAS growing in their native (US) and invaded (SA) environments. For all three studied species, we observed greater diversity of major metabolites in SA samples in comparison to the US samples. Increased metabolomic diversity in the invasive regions suggest that IAS may employ metabolic flexibility and/or rapid, adaptive evolution to succeed as alien invaders. Metabolic flexibility may be important to counteract different stresses, pathogens and herbivores encountered at KNP, compared to native habitats in the US and to successfully compete, and often replace, local flora. Interestingly, for *D. stramonium*, we observed that even the different sampling locations in the KNP are reflected in the differences in metabolomic profiles (Fig. 2C). Comparing the PLS-DA plots for the three species in this study, *D. stramonium* in SA showed the largest 95% confident region, suggesting greater metabolomic diversity than the other two species. At present, the role of individual metabolites and their significance to invasive behaviors is unclear. However, it is tempting to speculate that the observed changes relate to the global invasive success of *D. stramonium* and other IAS. This suggestion is supported by the fact that major secondary metabolites identified in the studied plants play a particularly important role in defense against biotic and abiotic stresses. These metabolites are flavone glycosides, flavones, and phytoprostanes (Table 1); polyphenols, sesquiterpenes, sesquiterpene lactones (Table 3) and alkaloids and steroids (Table 4).

Plants often respond and adapt to their environment by qualitatively and quantitatively changing their metabolomic profiles^11^ in a process often referred to as elicitation^28^. Secondary products involved in defense against pathogens (phytoalexins)^29^ or herbivores (antifeedants**)**^30^ may be elicited to higher levels or *de*-*novo* synthesized in response to perceived stresses or environmental changes. It is reported that flavonoids, abundant in *C. odorata*^31^, sesquiterpene lactones, abundant in *X. strumarium*^32^, and tropane alkaloids, abundant in *D. stramonim*^33^, represent groups of compounds involved in defense against pathogens and herbivory. It is not clear whether the observed statistical differences in the biochemical composition between US and SA populations represent a) inherited genetic changes related to natural selection; b) reflect reversible epigenetic adaptations to environmental stresses; or c) are just coincidental with invasive behavior. Growing US and SA plants side by side in a controlled environment may begin to answer this question. It was also suggested that successful IAS may share defense-related metabolites with their native neighbors^31^, possibly due to shared stresses experienced in a the same location. The methods described in this manuscript may help address this intriguing possibility.

Due to the high variability in the IAS metabolome, the metabolomic analysis described in this manuscript provides a wealth of structural information and unique metabolomic fingerprints that can be used for taxonomic identification of species or, as Fig. 2 suggests, for separating geographically isolated populations of the same species. We use PLS-DA score plots to visualize metabolome differences. In general, the PLS-DA score plots may represent an overoptimistic demonstration of group-wise difference in the metabolomic profiles when the number of measurements greatly exceeds the number of samples^34^. In our study, we focus on the most abundant metabolites in each plant species. The number of metabolites is smaller than 2 times the number of samples, therefore the over-fitting risk is small. Furthermore, group-wise separation is validated by the PERMANOVA tests. PLS-DA score plots are also used to reveal outlier samples and location-specific patterns. The area in the PLS-DA plots also visualize the degree of metabolomic variability in natural and invaded habitats. Therefore, metabolomic approaches, similar to those used in this investigation, may be a valuable supplementation to morphological and genomic/sequencing methods widely used in modern taxonomy and phylogeny. Limitations to the metabolomic approach to taxonomy may be the lack of species-specific reference libraries of plant metabolomes, produced with a standard collection and analytical method. These will need to be accumulated over time.

Our findings are consistent with the notion that metabolic (biochemical) flexibility may be an important factor enabling successful invasive behavior in plants. Combination of RAMES sampling methods, UPLC-MS profiling and PLS-DA and PERMANOVA statistical evaluation demonstrated that plants in the invaded habitats exhibited statistically significant metabolomic differentiation from their native habitats and had greater phytochemical diversity. Data collection and processing methods employed in this investigation were sensitive enough to differentiate species as well as geographically separated populations within each species. Overall, the presented data helps our understanding of how metabolomic profiles of IAS change depending on their temporal and spacial separation, and provides a valuable new approach to taxonomic analysis.

## Supplementary information

Supplementary information.
