# Supplementary material for: Metabolomic differences between invasive alien plants from native and invaded habitats
Source: Sci Rep. 2020 Jun 16;10:9749. doi: 10.1038/s41598-020-66477-w (PMC7297986; doi:10.1038/s41598-020-66477-w)
Supplement: Supplementary file 1 — Supplementary information. [file 41598_2020_66477_MOESM1_ESM.pdf]

Metabolomic differences between invasive alien plants from native and invaded habitats

Short title: Metabolomic profiling of invasive alien species

Sarah A. Skubel[1], Xiaoyang Su[2], Alexander Poulev[1], Llewellyn C. Foxcroft[3], Vyacheslav Dushenkov[4], Ilya Raskin[1*]

[*]raskin@rutgers.edu

1 Department of Plant Biology, Rutgers, The State University of New Jersey, New Brunswick, New Jersey, United States of America, **2** Department of Medicine, Robert Wood Johnson Medical School, Rutgers, The State University of New Jersey, New Brunswick, New Jersey, United States of America, **3** Centre for Invasion Biology, Department of Botany and Zoology, Stellenbosch University and Scientific Services, South African National Parks, South Africa **4** Hostos Community College, City University of New York, Bronx, New York, United States of America

Supplementary Information

S1 Table. Collection details on samples collected for the purpose of this study including country of origin, common and Latin names, date, GPS coordinates and collector initials (SS: Skubel, Sarah; RI: Raskin, Ilya, PhD). Identifier numbers shown are continued from samples previously collected but unrelated to this study.

ID	Country	Common Name	Latin Name	Date Collected	Coordinates	Collected By
10-13	US	Blue Mistflower	*Chromolaena odorata*	5/30/2018	27°56'7"N, 81°34'29"W	SS
14-16				5/30/2018	27°56'4"N, 81°34'42"W	SS
17-19				5/30/2018	27°56'21"N, 81°34'42"W	SS
20-23				5/30/2018	27°56'21"N, 81°34'42"W	SS
24-27		Rough Cocklebur	*Xanthium strumarium*	6/27/2018	40°47'57"N, 73°55'11"W	SS
28-31				6/27/2018	40°48'23"N, 73°55'33"W	SS
32-35				6/27/2018	40°47'57"N, 73°55'10"W	SS
36-38		Jimsonweed	*Datura stramonium*	6/27/2018	40°47'7"N, 73°56'7"W	SS
39-42				6/27/2018	40°46'59"N, 73°56'41"W	SS
43-46				7/02/2018	40°27'27"N, 74°24'35"W	SS
50-53	SA	Blue Mistflower	*Chromolaena odorata*	7/24/2018	24°58'9"S, 31°35'38"E	SS & RI
54-58		Jimsonweed	*Datura stramonium*	7/24/2018	24°58'9"S 31°35'38"E	SS & RI
59-61				7/25/2018	24°59'1"S, 31°38'33"E	SS & RI
62-67		Rough Cocklebur	*Xanthium strumarium*	7/26/2018	24°57'26"S, 31°42'56"E	SS & RI
68-74				7/28/2018	23°73"S, 31°27'22"E	SS & RI
75-81				7/28/2018	23°8'41"S, 31°27'44"E	SS & RI
82-88				7/28/2018	23°6'30"S, 31°26'16"E	SS & RI

89-99	SA	Blue Mistflower	*Chromolaena odorata*	7/30/2018	22°25'31"S, 31°15'17"E	SS & RI
100-107				7/31/2018	22°25'21S, 31°14'0"E	SS & RI
108-115				7/31/2018	22°25'19"S, 31°14'59"E	SS & RI
116-123				7/31/2018	22°25'20"S, 31°14'46"E	SS & RI
124-130		Rough Cocklebur	*Xanthium strumarium*	7/31/2018	22°25'21"S, 31°14'40"E	SS & RI
131-138				8/3/2018	23°45'27"S, 31°22'17"E	SS & RI
139-144				8/3/2018	23°45'14"S, 31°22'0"E	SS & RI
145-152		Jimsonweed	*Datura stramonium*	8/3/2018	23°45'14"S, 31°22'0"E	SS & RI
153-160				8/6/2018	23°40'55"S, 30°59'44"E	SS & RI
161-168				8/6/2018	23°40'18"S, 30°59'20"E	SS & RI

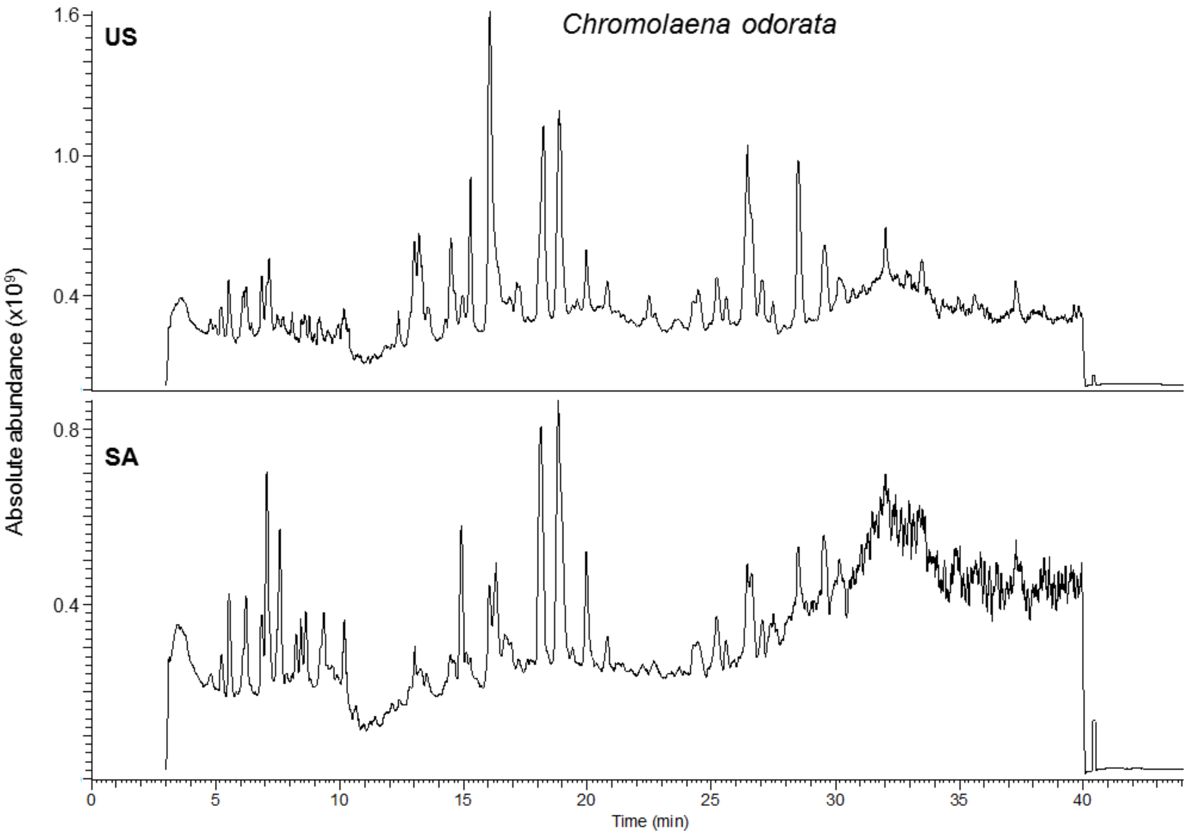

S2 Fig. 1. (-)ESI MS total ion current chromatograms [m/z 100 – m/z 1000] of disk eluates from *Chromolaena odorata* extracts from plants originating in the United States (US), and South Africa (SA).

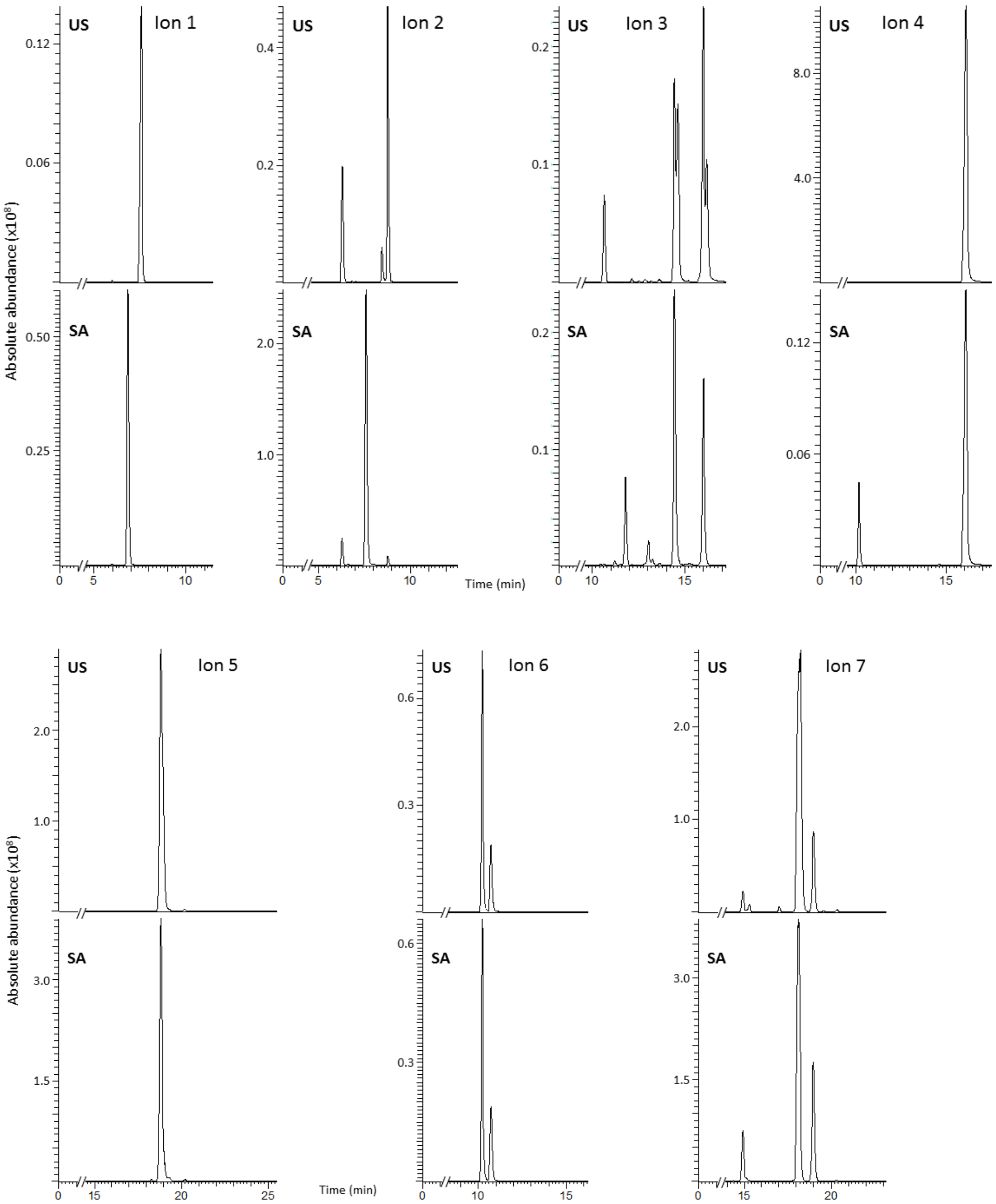

S2 Fig. 2. (-)ESI MS extracted ion current chromatograms of disk eluates from *Chromolaena odorata* extracts from plants originating in the United States (US), and South Africa (SA). Ion number details – see Table 1.

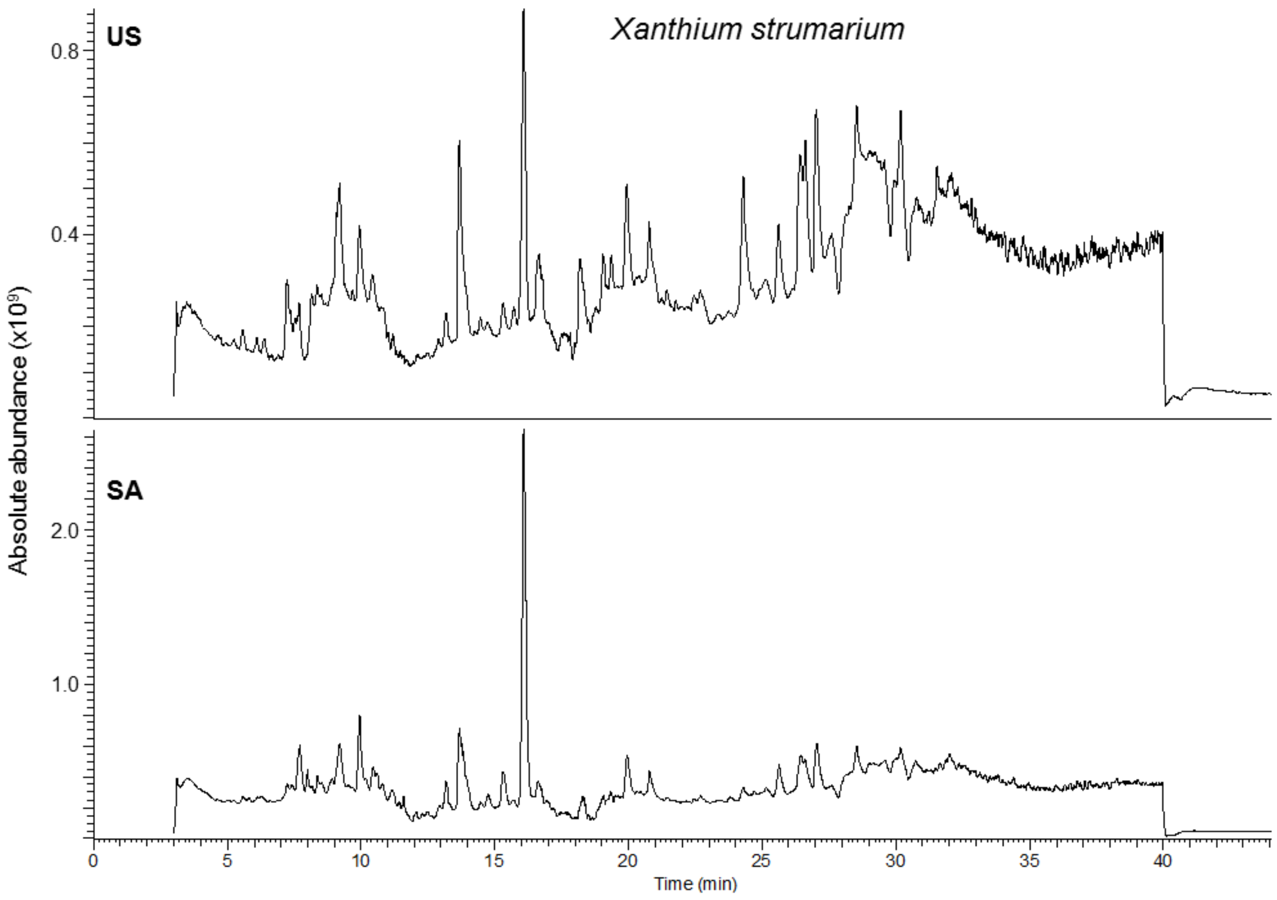

S2 Fig. 3. (-)ESI MS total ion current chromatograms [m/z 100 – m/z 1000] of disk eluates from *Xanthium strumarium* extracts from plants originating in the United States (US), and South Africa (SA).

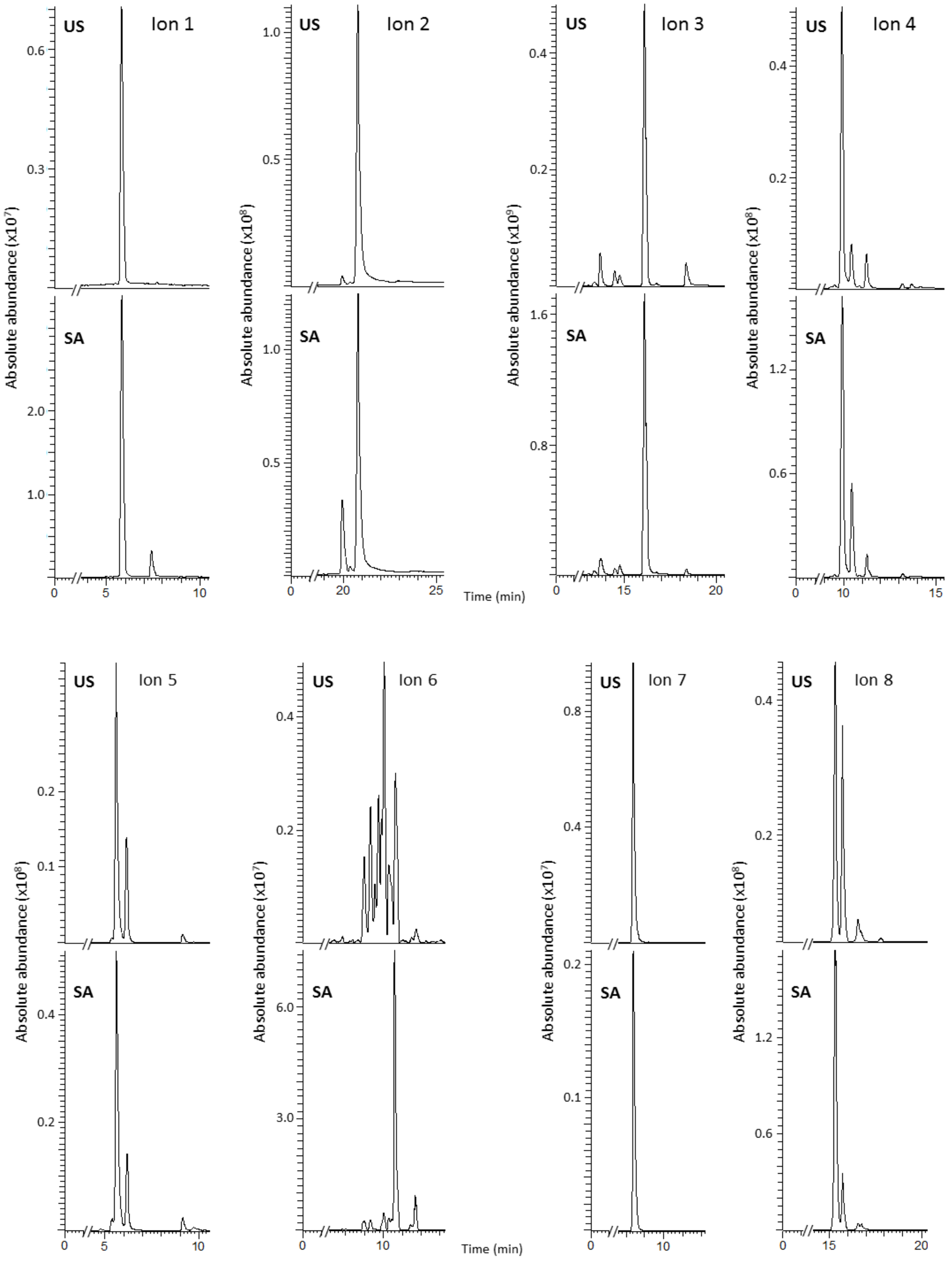

S2 Fig. 4. (-)ESI MS extracted ion current chromatograms of disk eluates from *Xanthium strumarium* extracts from plants originating in the United States (US), and South Africa (SA). Ion number details – see Table 2.

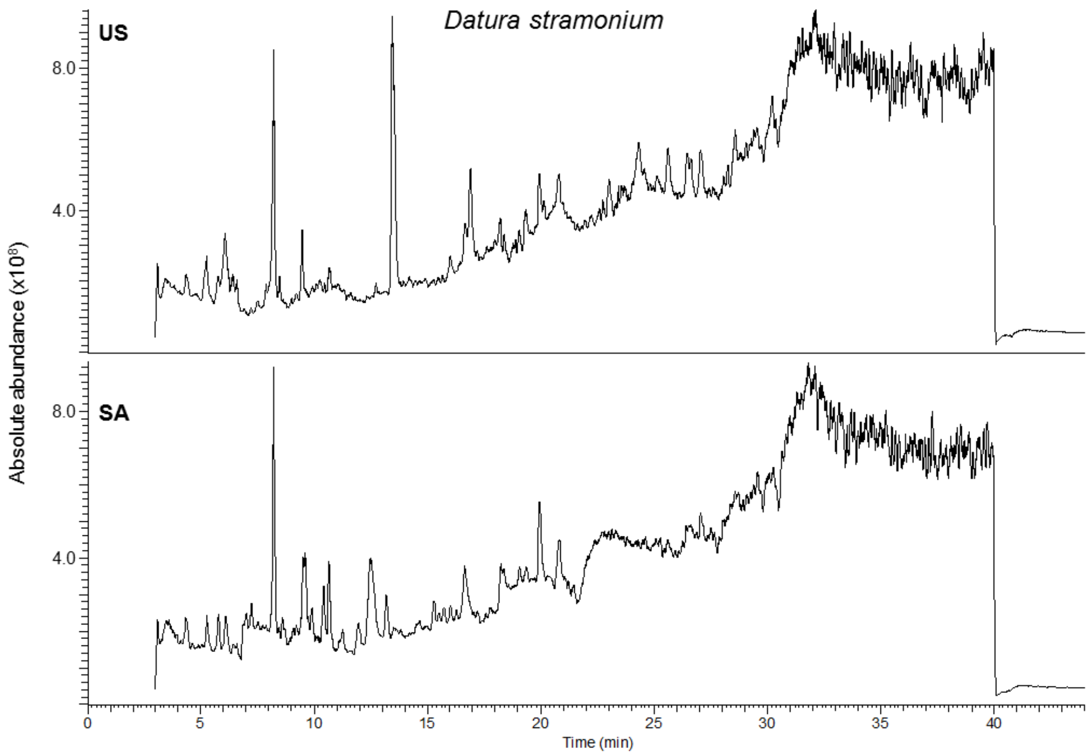

S2 Fig. 5. (-)ESI MS total ion current chromatograms [m/z 100 – m/z 1000] of disk eluates from *Datura stramonium* extracts of plants from the United States (US), and South Africa (SA).

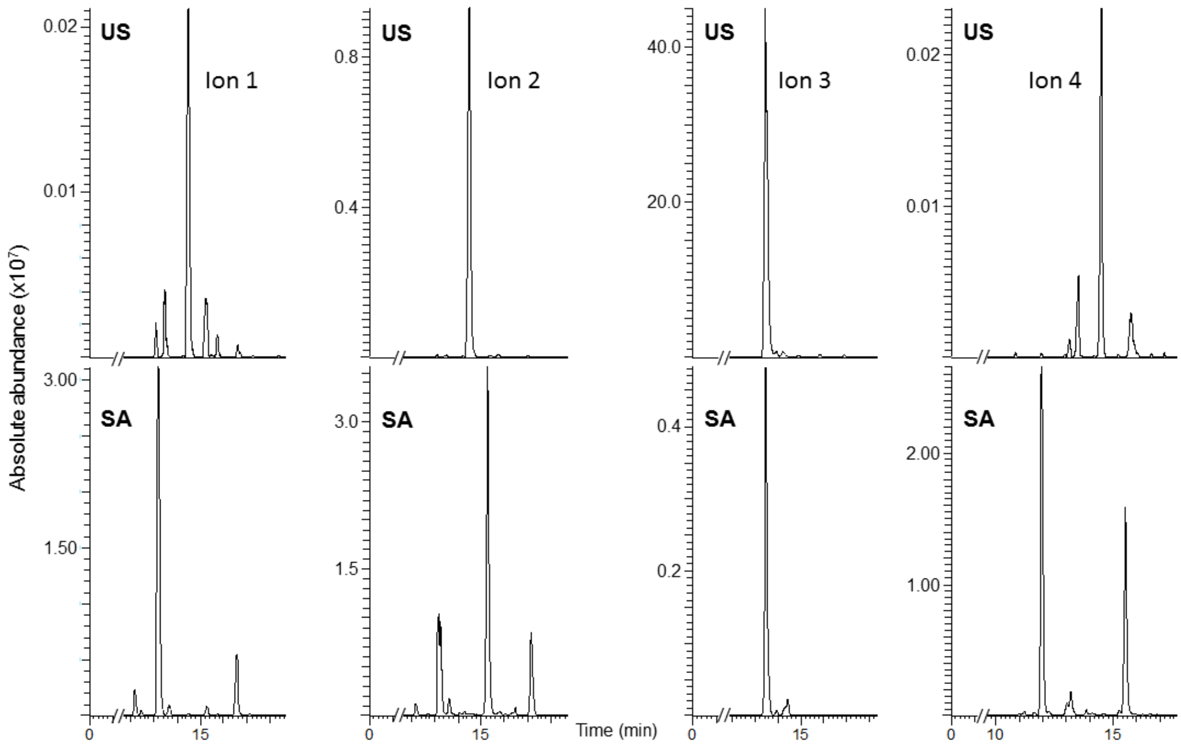

S2 Fig. 6. (-)ESI MS extracted ion current chromatograms of disk eluates from *Datura stramonium* extracts from plants originating in the United States (US), and South Africa (SA). Ion number details – see Table 3.

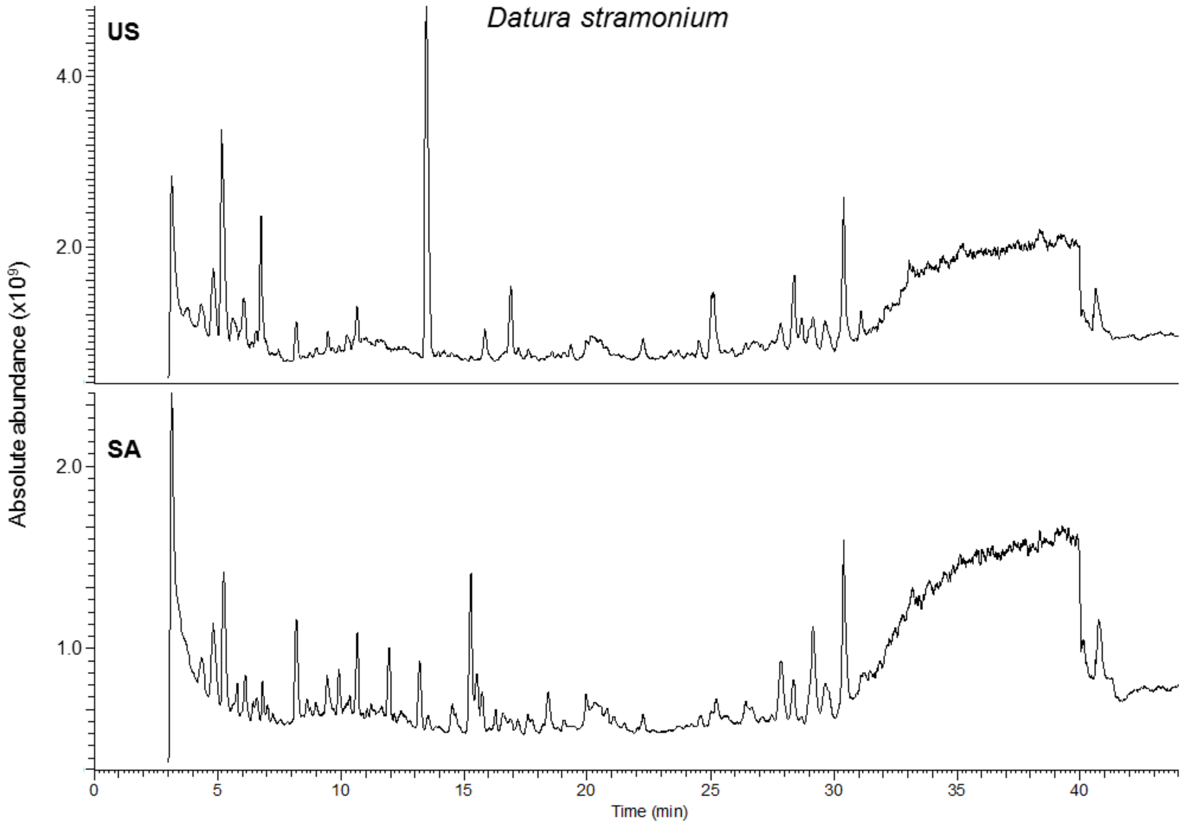

S2 Fig. 7. (+)ESI MS total ion current chromatograms [m/z 100 – m/z 1000] of disk eluates from *Datura stramonium* extracts of plants from the United States (US), and South Africa (SA).

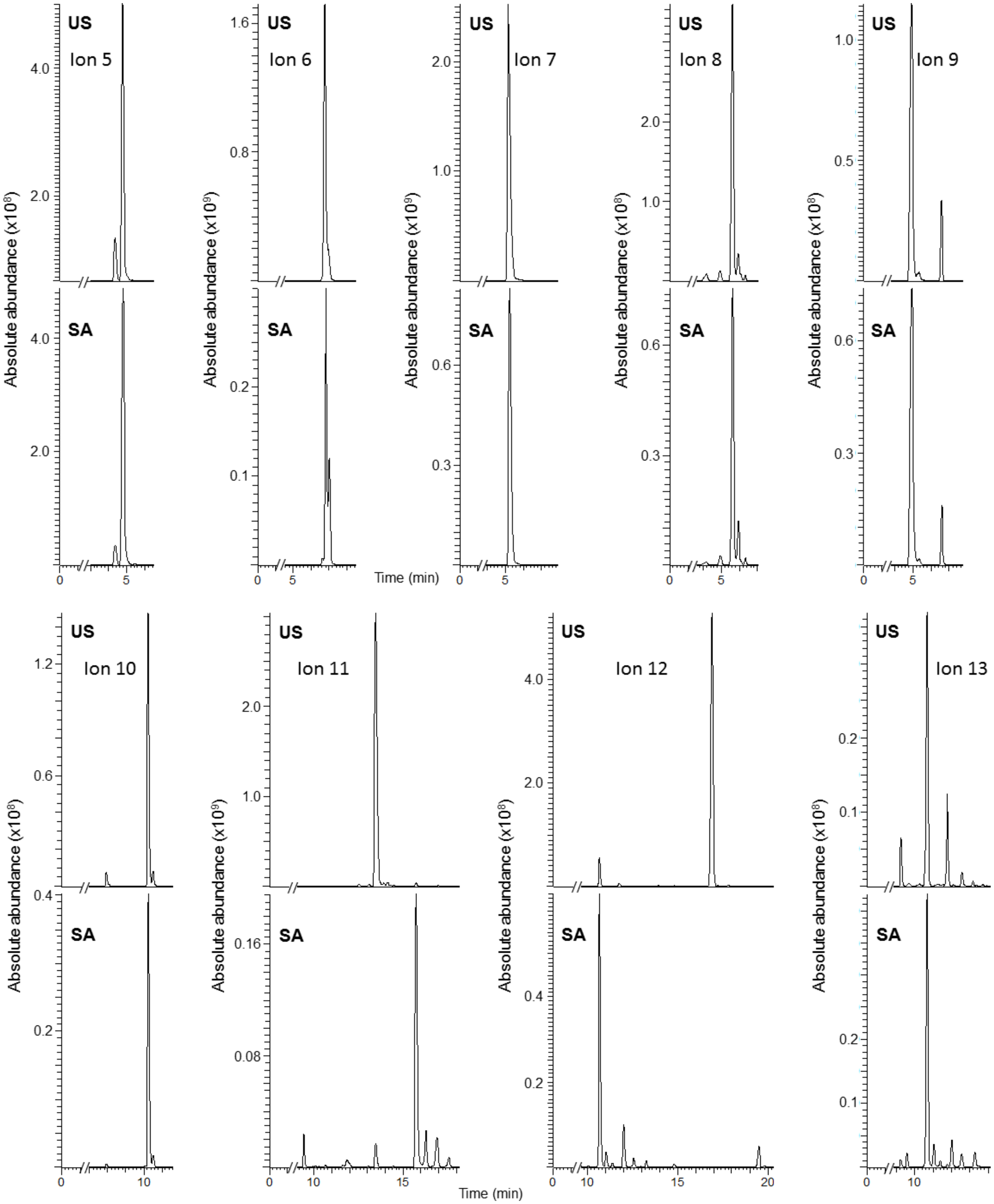

S2 Fig. 8. (+)ESI MS extracted ion current chromatograms of disk eluates from *Datura stramonium* extracts from plants originating in the United States (US), and South Africa (SA). Ion number details – see Table 3.
